# Modeling of two-stage anaerobic onsite wastewater sanitation system to predict effluent soluble chemical oxygen demand through machine learning

**DOI:** 10.1038/s41598-023-50805-x

**Published:** 2024-01-21

**Authors:** Rajshree Mathur, Meena Kumari Sharma, K. Loganathan, Mohamed Abbas, Shaik Hussain, Gaurav Kataria, Mohammed S. Alqahtani, Koppula Srinivas Rao

**Affiliations:** 1 Department of Civil Engineering, Manipal University Jaipur, Jaipur, 303007 Rajasthan India; 2 Department of Mathematics and Statistics, Manipal University Jaipur, Jaipur, 303007 Rajasthan India; 3 Electrical Engineering Department, College of Engineering, King Khalid University, 61421 Abha, Saudi Arabia; 4 Trenchless Technology Center (TTC), Louisiana Tech University, Ruston, USA; 5 Department of Chemical Engineering, Manipal University Jaipur, Jaipur, 303007 Rajasthan India; 6 Radiological Sciences Department, College of Applied Medical Sciences, King Khalid University, 61421 Abha, Saudi Arabia; 7 BioImaging Unit, Space Research Centre, Michael Atiyah Building, University of Leicester, Leicester, LE1 7RH UK; 8 Department of Computer Science and Engineering, MLR Institute of Technology, Hyderabad, Telangana India

**Keywords:** Biogeochemistry, Environmental sciences, Chemistry, Engineering, Mathematics and computing

## Abstract

The present research aims to predict effluent soluble chemical oxygen demand (SCOD) in anaerobic digestion (AD) process using machine-learning based approach. Anaerobic digestion is a highly sensitive process and depends upon several environmental and operational factors, such as temperature, flow, and load. Therefore, predicting output characteristics using modeling is important not only for process monitoring and control, but also to reduce the operating cost of the treatment plant. It is difficult to predict COD in a real time mode, so it is better to use Complex Mathematical Modeling (CMM) for simulating AD process and forecasting output parameters. Therefore, different Machine Learning algorithms, such as Linear Regression, Decision Tree, Random Forest and Artificial Neural Networks, have been used for predicting effluent SCOD using data acquired from in situ anaerobic wastewater treatment system. The result of the predicted data using different algorithms were compared with experimental data of anaerobic system. It was observed that the Artificial Neural Networks is the most effective simulation technique that correlated with the experimental data with the mean absolute percentage error of 10.63 and R^2^ score of 0.96. This research proposes an efficient and reliable integrated modeling method for early prediction of the water quality in wastewater treatment.

## Introduction

AD is a highly sensitive process therefore different control parameters like environmental emission, energy consumption, digestion procedure and feed pattern are very important to optimize the treatment process and performance efficiency. Chemical Oxygen Demand (COD) is one of the most important controlling parameters that effect the operation of anaerobic treatment unit. Effluent COD is often expressed as a demand or constraint parameter, as higher COD levels not only affect biogas production efficiency but are also hazardous for the ecosystem. Therefore, a close check on the value of COD is essential. Predicting the value of effluent COD has remained a primary research concern and demands extensive knowledge and understanding of the complex biochemical processes involved in anaerobic digestion.

Due to the complex process of anaerobic digestion, use of mathematical modeling is recommended and fundamental models of varying complexities describing anaerobic digestion process have been developed in the last four decades^1,2^. Anaerobic reactors are trending due to the improvements in extensions and structure with the help of modeling tools such as ADM simulation^3^. However, it is essential to develop model further with more optimization and control strategies due to large instability observed in the anaerobic digester operation^4^. Techniques to deliver the control strategies and estimation of complex dynamics for the treatment of wastewater can be found in several research works^5^. Anaerobic digestion model No. 1 or ADM1^6^ explains the anaerobic digestion process in two continuous reactors with identification and reduction of complex dynamics through neural network method. This method developed a stabilizing optimal control strategy for the production of methane and hydrogen in a desired way.

Prediction is a primary tool of artificial intelligence (AI) technology in a variety of areas. AI subset machine learning (ML) identifies patterns in data for prediction or classification purposes^7^. AI approaches are being used to describe and predict environmental events due to their high precision compared to mechanical models^8^. These algorithms are more effective in learning complex associations than statistical methods. This is accomplished by using an ANN model that is fully connected, and each neuron in the network has trainable parameters (weights and biases). Feedforward RNA can be employed for wastewater or sewage treatment plant quality prediction. To simulate the influent or effluent wastewater parameters, several studies have been carried out. For example, ANN models are used to estimate methane production in a biogas optimization scenario with (R^2^ = 0.87)^9^. In addition, another similar modelling study was undertaken to determine the association between the addition of membrane bioreactor additives and the WWTP. It was found that the hybrid genetic algorithm with fuzzy logic (GA-FIS) model was more accurate than the fuzzy logic (ANFIS) model at predicting missing values in wastewater parameters, such as COD, BOD, and NH_4_-N. In contrast to ANFIS prediction, integrated GA-FIS demonstrated smaller errors^10^. An ELM model paired with kernel principal component analysis (KPCA) was used by Abba et al. in another investigation to predict pH, turbidity, total suspended particles, and hardness with the highest accuracy (R^2^ > 0.95)^11^. Random forest (RF) and gradient boosting (GBM) approaches are other excellent ML methods that are at the cutting edge of technology. Small WWTPs in the UK were found to benefit from the application of an RF prediction model^12^. Another pre-processing approaches and selection of various features of digestion system, increases the training speed, improves prediction accuracy, and simplifies the models^13^. Most forecasting studies, however, use correlation models, such as the Pearson correlation approach. Due to this, it is still necessary to compare the effects of FS and other simulation methods for WWTP components. Other machine learning techniques, such as ANN, SVM, etc. are more commonly employed to predict WWTP components^14,15^. The following difficulties occurred in creating the mathematical model for the anaerobic digestion (AD) process:

- It is not feasible to build a simple mathematical model because of the intricate processes involved.
- Another difficult task is defining a particular mathematical equation to capture the intricate physicochemical process.
- Such an approach also precludes data visualization and the impact of process factors on the outcome.

Artificial intelligence (AI) approaches are being explored to address the limits of anaerobic digestion modelling in light of the aforementioned constraints. AD involves complex metabolic pathways, diverse microbial communities, and influence of only a certain type of microbial communities on the digester performance. Due to this, ML is a promising solution for predicting the process parameters.ML process, if implemented correctly on a quality training data, can help design and process engineers in efficient decision making. Additionally, ML models are easier to understand and improved as compared to mathematical models as ML is a data driven approach and selects the user from the inherent process complexities.

The aim of this research is to develop machine learning models for forecasting effluent COD. The developed model was tested by using the data collected from a two-stage digester over a period of one year. The rest of the paper is organized as follows: Previous research works are discussed in Section II. Material and Methodology are discussed in section III. The results and discussion are demonstrated in Section IV. Section V concludes the research and highlights possible future directions.

## Literature review

In recent years, mathematical modeling has played a significant role in the design, optimization, and control of various AD processes. The most widely used model is Anaerobic Digestion Model No. 1 (ADM1), developed by the IWA Task^16^. This model was basically created to simulate AD sewage sludge. It has been observed that modeling of anaerobic digestion is a highly complex task due to the nonlinear relationships between input and target variables. Forecasting and optimization also make use of ADM simulation. Other benefits of modelling anaerobic digestion include increased flexibility and simple problem detection^17^. Numerous aquatic modelling programs are available that can be verified on many platforms, including AQUASIM, SIMBA, MATLAB, GPSX, and WEST. The ADM1, Task Group for MATLAB, and AQUASIM are responsible for developing the most widely used tools for AD implementation^18–20^. Specific numerical approaches are needed for every implementation of these simulation schemes. Cross validation or hybrid approaches are often preferred to overcome the limitations of single model. Anaerobic Digestion Model No.1 (ADM1), developed by the IWA Task Group for Mathematical Modelling, is a benchmark model for simulation of anaerobic digestion^21^. ADM1 consists of all four comprehensive stages of anaerobic digestion as mentioned in the previous section and a preprocessing stage. The model is used to identify various input and output parameters such as COD based on 32 ordinary differential equations using COD concentration only. ADM1 involves decoupling of lumped variables, which signifies that most of the variables are solved independently. It can be observed that developing a system of straightforward mathematical equations that can accurately represent all the physiochemical processes involved in AD is not only a strenuous task but also leads to the loss of information. Furthermore, data visualization and impact of input parameters on output cannot be addressed by using this approach.

Due to the limitations mentioned above, Machine Learning (ML) techniques have been adopted in the present study. ML is a promising solution for predicting the process parameters. ML, if implemented correctly on quality training data, can help design and process engineers in making efficient decisions. Additionally, ML models are easier to understand and can be improvised as compared to mathematical models. This is because ML is a data-driven approach, and the user can build an efficient ML model without much understanding of the inherent process complexities^22^. Recent improvements in computing power have made it possible to develop Machine Learning as a model development tool for pattern recognition, statistics, and optimization.

Clercq et al.^23^ used various machine learning algorithms to predict the performance of an anaerobic digester based on fluctuating values of bio waste input. The machine learning models used in the research were Tree Boosting Algorithm, K Nearest Neighbors, Random Forest, and Logistic regression. Similarly, hybrid algorithm of artificial neural network and genetic algorithm was used to optimize anaerobic digestion^24^. Baek et al.^25^ used various machine learning based models for predicting anaerobic digestion performance.

The artificial neural networks were used to predict the changes in the composition of the microbial community because of environmental stresses^26^. Some studies have been conducted over a period of time bycombining regression analysis and artificial neural networks to evaluate the performance of wastewater treatment plants in Iran^27^.

Artificial Neural Networks (ANN) was also used for simulation of the up-flow anaerobic sludge blanket process. The process is highly unstable and is vulnerable to load fluctuations. The organic content of the effluent substrate was predicted using ANN. Experimental results demonstrated that the predicted values were in good relationship with the actual values^28^. Nair et al.^29^ used back propagation ANN to determine the impact of changes in organic loading rate and type of substrate (food waste, vegetable waste, and yard trimmings) on methane formation. From the literature^30^, it can be concluded that ANN is highly suitable for the simulation of anaerobic digestion and forecasting process parameters.

There are certain challenges while using ANN, such as ANN being a black box model requires extensive training data. Also, ANN cannot efficiently identify patterns or relationships in case of noisy or unstructured data. Other machine learning tools, such as tree-based pipeline optimization tool used to develop an improved understanding of different waste inputs and operating conditions, which impact biogas yield^31^.

Despite the limitations and availability of other mathematical models, ANN is the most widely used technique for predicting process parameters in AD. There is a dearth of research that tests the usability of other ML models, such as Regression models and Ensemble Learning techniques, in the given context. In the present study, ANN was compared to primary ML models (Decision tree and linear regression) as well as ensemble learning (Random Forest), to identify the most suitable approach for SCOD (soluble chemical oxygen demand) prediction.

After studying the research done in the past, it has been found that till now, no work has been done on the simulation modeling of effluent characteristics of a two-stage anaerobic onsite wastewater sanitation system, which is a combination of two different types of suspended and attached growth treatment processes within a single unit. The aim of this research was to develop the machine learning models for forecasting effluent SCOD of domestic wastewater after treatment using two-stage anaerobic onsite sanitation system. The developed model was tested by using the data collected from a two-stage digester over a period of one year.

## Material and methodology

### Anaerobic system and data collection

**Figure 1:**
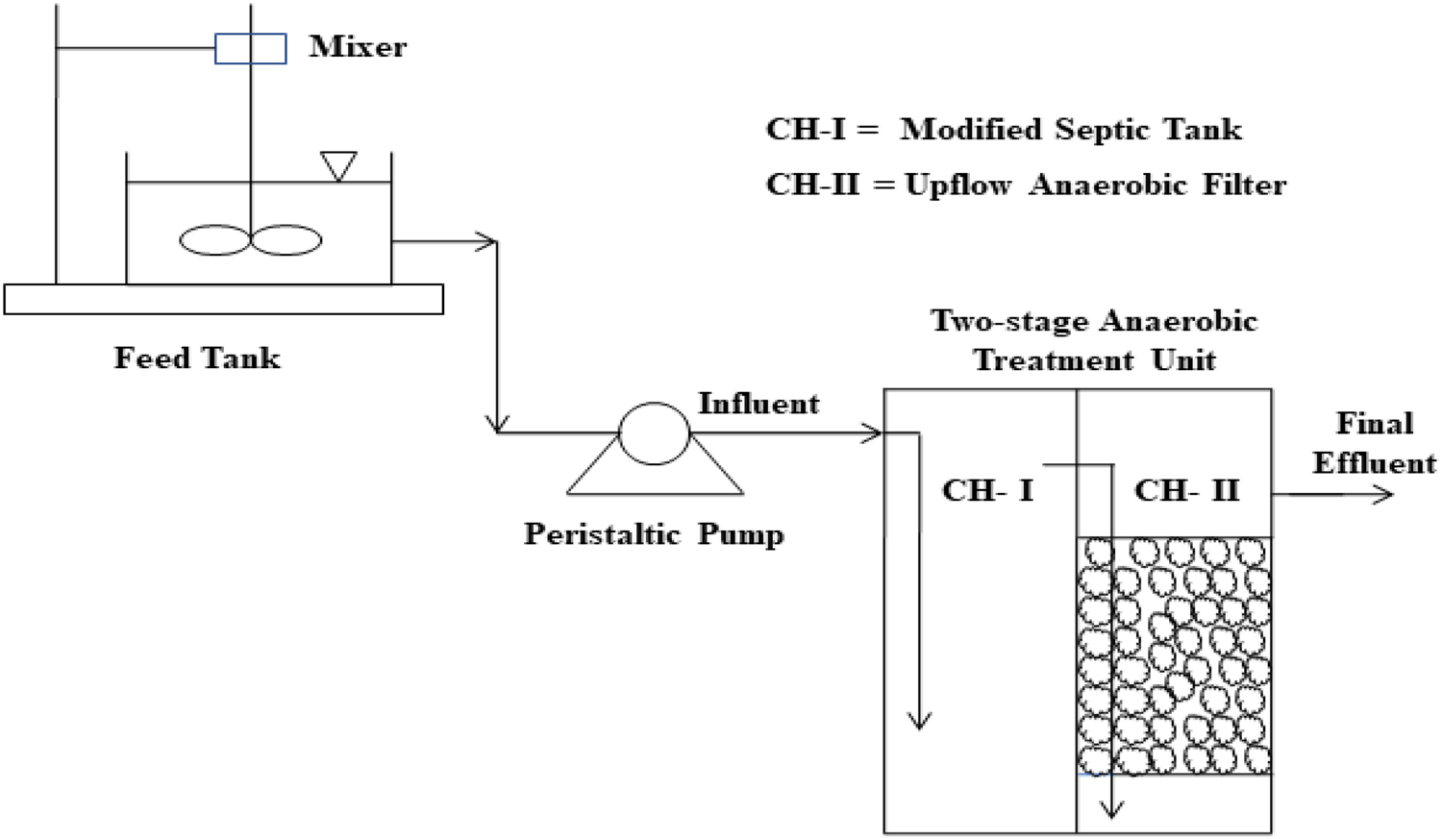
Line diagram of two-stage anaerobic treatment reactor.

The data for the present study was obtained from a two-stage laboratory scale anaerobic treatment reactor installed at Jaipur, India^32^. Figure 1 shows the reactor used in the present study and its experimental setup. The two-stage reactor consisted of two semi-cylindrical chambers, created by dividing a single cylindrical unit into two parts as illustrated in Fig. 1. The first chamber worked as a modified septic tank and the second one as an anaerobic filter to perform post-treatment of the modified septic tank effluent. The second chamber was randomly packed with media and fed with the effluent of the first chamber, which entered it from the bottom. The media (baked clay of 20 mm size) was kept on a semi-circular perforated plastic plate, placed at a height of 60 mm from the bottom of the tank. The total effective volume of the two-stage system was 24 L having unit dimensions of 450 mm of height and 300 mm of diameter.

All experiments were carried out in the two-stage anaerobic treatment reactor under different operating conditions. The concentrations of all parameters of actual domestic wastewater injected into the system varied greatly. The system was started without vaccination and was operated for a long time. Monitoring during the study period was carried out at temperature range of 17–45 °C. The efficiency of anaerobic treatment was examined at different hydraulic and organic loading rates.

The anaerobic two-stage treatment unit was continuously fed with water at a constant flow rate of 24 L/day, equal to 24 h hydraulic retention time (HRT), until the system reached steady state. All wastewater resulting from domestic activities flowed directly to the treatment facilities without changing its properties. It was then fed with domestic wastewater that was collected on a daily basis. For a week, the reactor was maintained in anaerobic conditions. After that, it was fed with household wastewater that collected on daily basis.

**Figure 2:**
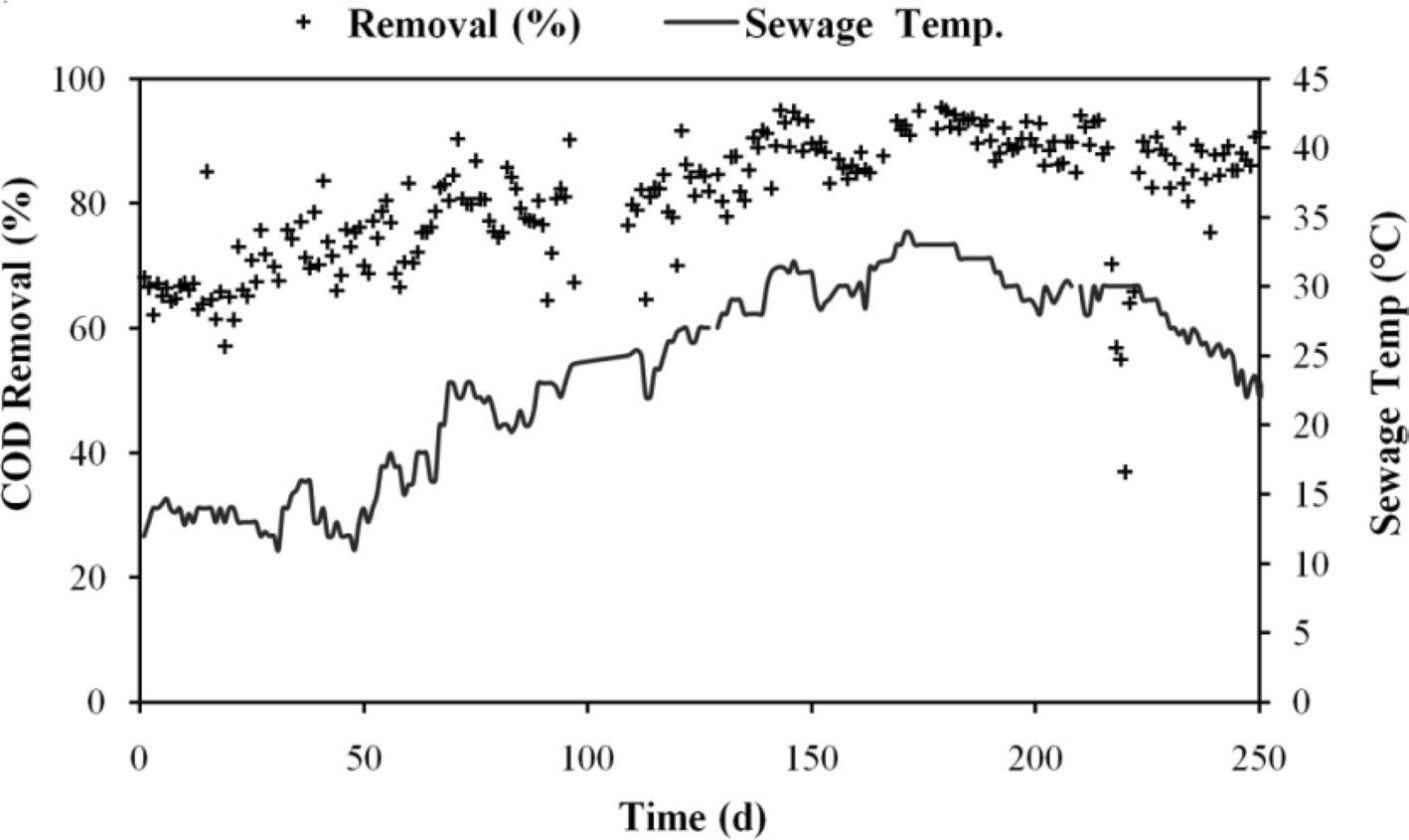
Changes in COD elimination effectiveness with temperature.

Temperature was found to have an impact on the anaerobic biodegradation of organic matter over the research period. Wastewater temperature was found to change during the day by 11–49 °C. Figure 2 shows how temperature changes affect the effectiveness of COD removal. When the system was first started in the winter, it was noted that when the temperature rose over time, the system's total COD removal effectiveness improved. When sewage reached its maximum temperature of 45 °C, 92.5% COD removal effectiveness was recorded.

The input parameters were measured from wastewater drawn from the points of influent, and effluent parameters from the outlet of the secondary chamber. Following influent and effluent parameters were analyzed by standard procedure (APHA):

- Total alkalinity (T. Alkalinity (mg/L)),
- Influent chemical oxygen demand (inf CODT (mg/L))
- Influent soluble chemical oxygen demand (inf. SCOD (mg/L)),
- Total suspended solids (TSS (mg/L)),
- Influent Kjeldhal nitrogen (inf. TKN in mg/L)
- NH_3_^−^N (ammoniacal nitrogen (mg/L)) and
- NO_3_^−^N (nitrate nitrogen (mg/L).

### ADM1 performance

Modified ADM1 was developed according to two-stage anaerobic treatment reactor to predict effluent COD from modified ADM1. To accomplish this goal, the modelling and simulation of anaerobic digestion of household wastewater was conducted using elemental analysis and ADM1^33^.

Table 1 summarizes the reactors' liquid phases as well as the key ADM Model parameters that were employed in this investigation.

**Table 1:** ADM Model parameters.

S/no	Model parameters	Values
1	Temperature	11–49 °C
2	pH range	7.15–7.3
3	Total volume	0.024 m^3^
4	Volume of step 1 reactor	0.0171 m^3^
5	Volume of step 2 reactor	0.0069 m^3^

In accordance with the experimental setup, data has been collected for 365 days. The input parameters were measured from wastewater are drawn from the points of influent and effluent parameters from the outlet of the secondary chamber. Following influent and effluent parameters were analyzed by standard procedure (APHA):

Few assumptions made while developing the model are explained below.

- The concentration of input oxygen was assumed to be zero.
- Particulate substrate and inert particulate material were the only parameters considered to be present in primary wastewater.
- The influent included a reasonable amount of nitrogen.

The analysis's findings were converted to the appropriate units. The Matlab program developed for the elemental analysis approach that used the data as input. The stoichiometric coefficients for the empirical formula and the fractions of proteins, fats, carbohydrates, and volatile fatty acids (VFA) were the output results. The typical ADM1 model required a COD-based substrate concentration specification, but the equations for determining the substrate composition were expressed as C-molar fractions of the substrates. Based on converting fractions to COD, equivalent concentration was determined. For carbohydrates^34^, it is as follows:

$${\text{COD}}_{{{\text{CHO}}}} \left[ {{\text{gO}}_{{2}} {\text{dm}}^{{ - {3}}} } \right] \, = {\text{ TOC}} \cdot \eta_{{{\text{CHO}}}} \gamma_{{{\text{CHO}}}} /{4} \cdot {\text{ MWO}}_{{2}}$$

where the number 4 represented the number of electrons that were accepted per mole Oxygen and MWO_2_ was the molecular weight of oxygen. The findings of the simulation were compared to the COD measurements obtained from the two-stage reactor following the treatment of domestic wastewater.

**Figure 3:**
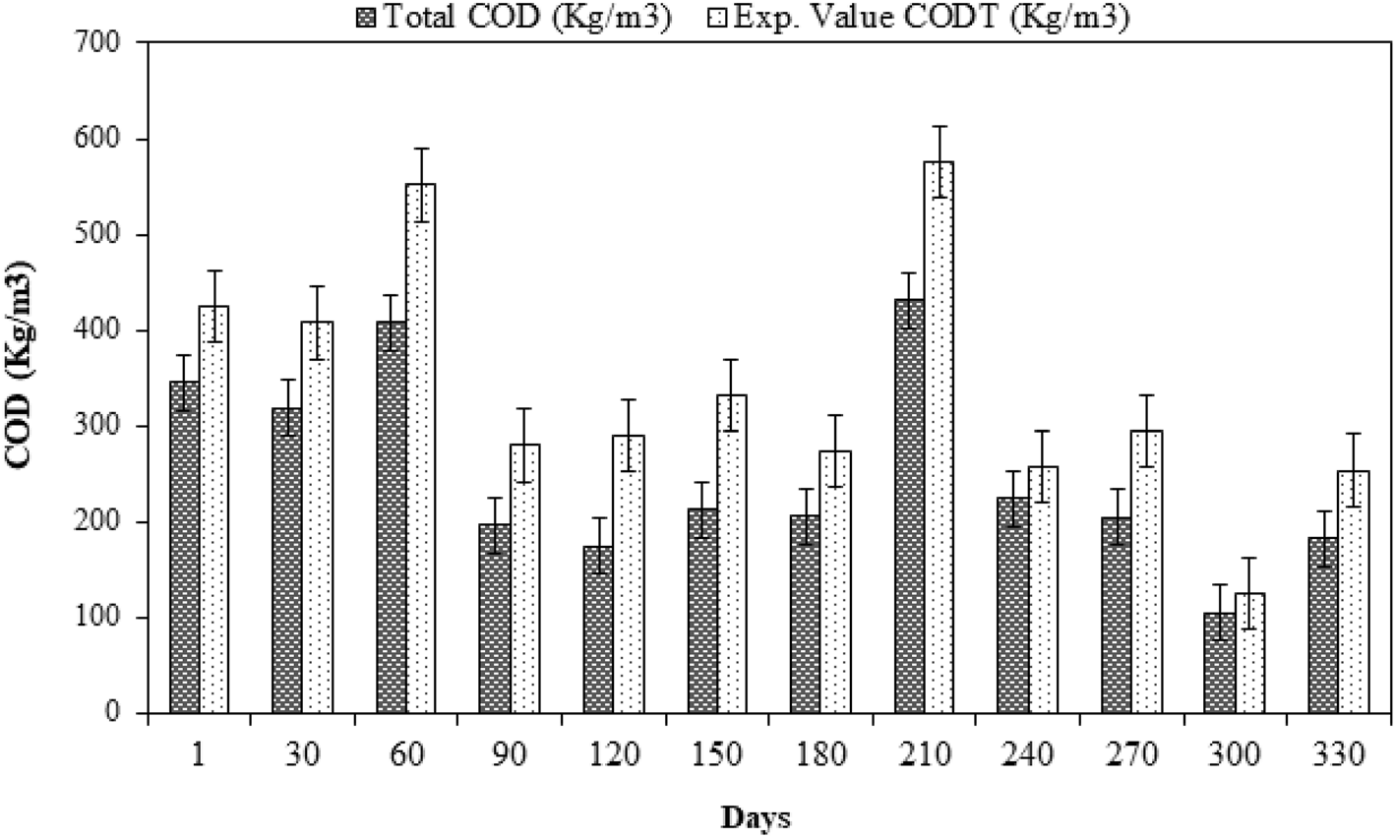
SCOD value variation from experiments with the ASM2ADM interface.

Based on the inlet concentration in raw domestic wastewater, a comparison of the measured and simulated effluent SCOD was also carried out (Fig. 3). The findings showed that there was a minor variation between the simulated and measured effluent data because the samples contained inorganic suspended particles also. The output from the ASM2ADM interface is displayed against the experimental SCOD as shown in Fig. 3.

Figure 3 illustrates the comparison between the measured SCOD and predicted SCOD values. The figure shows that the predicted values of SCOD closely matched with the measured values. To validate the results obtained from ADM1, machine learning techniques were used.

### Machine learning techniques

Four machine learning techniques, namely Linear regression, Decision Tree, Random-Forest, and Artificial Neural Networks (ANN) were used in the present study. The collected data for different input variables were normalized in accordance with Interquartile ranges. To enhance the quality of data, statistical techniques were applied leading to better classification through normalization.

#### Data pre-processing

Data pre-processing is the primary step that is performed for any data-driven analysis. For our purpose, the data normalization method was used. As the name implies, data normalisation is the process of improving the quality of data to improve classification. Redundancy and inconsistency must be eliminated by data normalisation. Null values were examined first for garbage in this study. These entries were removed from the database. By employing the equalisation histogram, the pre-processed database was normalised. Feature extraction and machine learning step were now possible. The data was then further refined for feature extraction and machine learning phase.

#### Feature selection

Initially, a correlation matrix is formulated to relate the input variables with each other. In this phase correlation matrix and mutual information gain are applied to filter out the most important features and remove duplicate features, if any. Correlation matrix denotes the correlation between different variables (input variables) for predicting target variable. High correlation denotes that the two variables affect the target variable similarly; therefore, one can be dropped from the analysis. Mutual Information Gain represents the significance or influence of input variables for predicting target variable. Input variables with high mutual information gain and less correlation were selected, and the others were discarded, thereby reducing computational overhead without affecting the performance. The steps involved in the process are listed below.

- Constant value features checking (Check 0 variance between the feature’s columns value).
- Check if there are any feature columns whose values are 99% same.
- Check feature importance or decide features’ ranking using correlation checking.
- Check features’ importance using mutual information gain.

**Figure 4:**
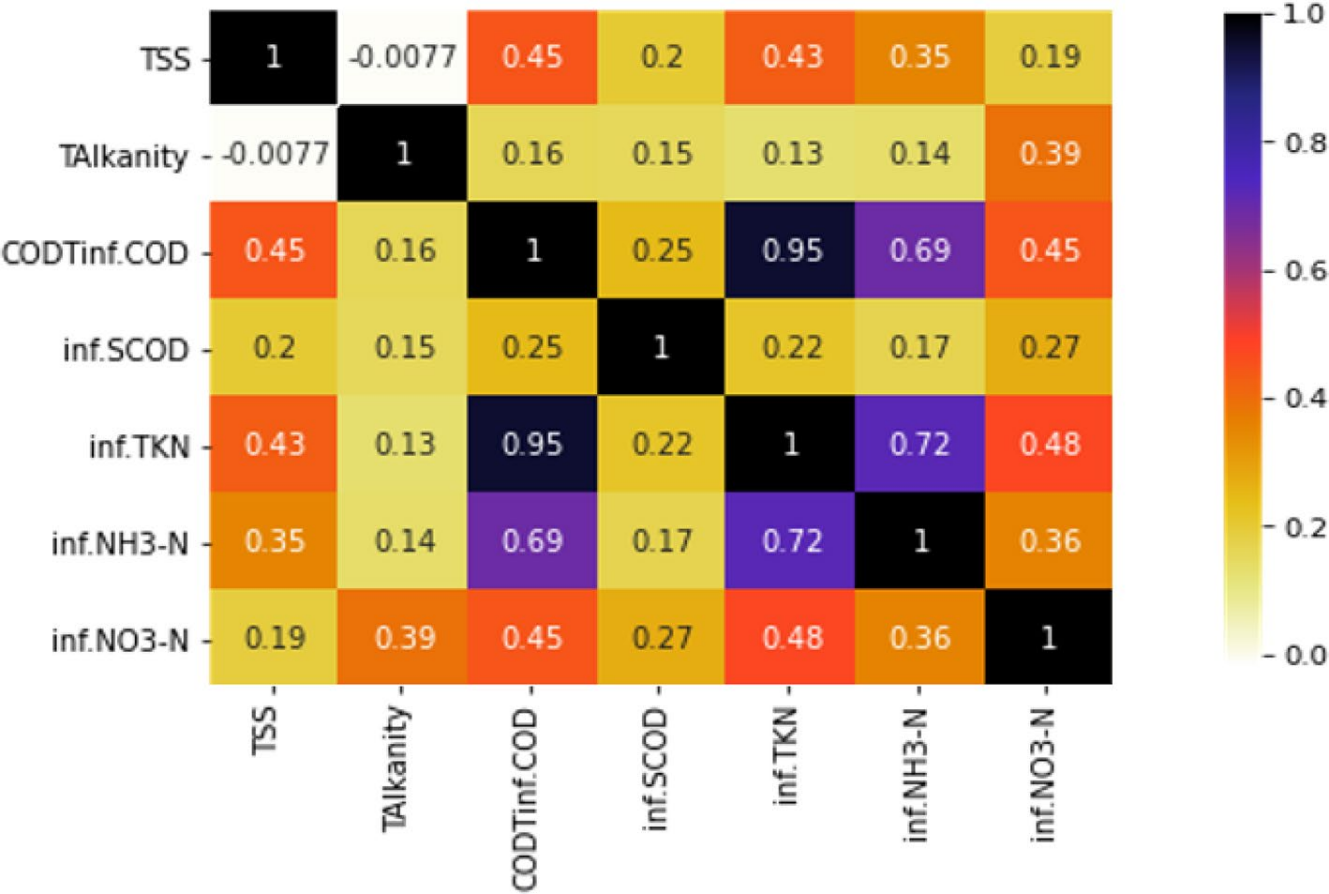
Correlation matrix of seven input variables.

**Figure 5:**
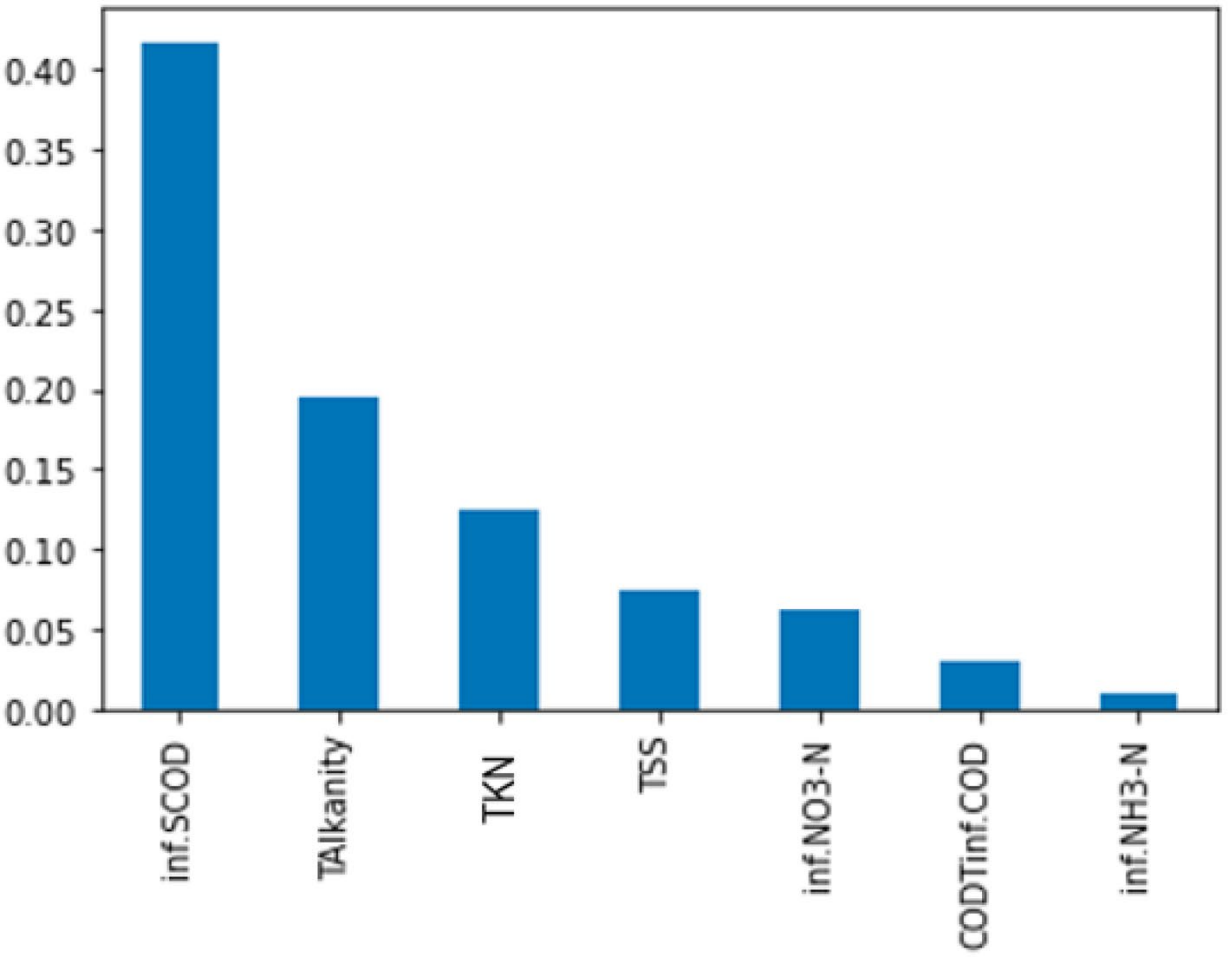
Mutual information gain value of the seven input variables.

Figure 4 represents the correlation matrix of the features, and Fig. 5 represents the importance of each feature based on mutual information gain.

It can be inferred that influent TKN and influent COD_T_ are 95% correlated. One can be assessed if the other parameter is known. Hence, for the study, influent TKN was omitted, and influent COD_T_ was considered. Table 2 gives the descriptive statistics of the final six input variables and Table 3 shows the descriptive statistics of the effluent soluble COD after normalization and Feature extraction.

**Table 2:** Descriptive Statistics of the data set used in the study.

S/no	Influent wastewater characteristics in mg/L	Minimum	Maximum	Mean	Standard deviation	Counts
1	SCOD	21.6	206.1	93.19	50.54	307
2	TSS	79.5	364.2	199.27	57.94	307
3	Total alkalinity	246	400	322.34	34.43	307
4	Ammoniacal nitrogen	1.6	44.3	19.73	7.25	307
5	Nitrate nitrogen	0.6	15.8	4.38	3.12	307
6	COD	87	780.7	350	120.96	307

**Table 3:** Descriptive Statistics of effluent wastewater characteristics used in the study.

S/no	Influent wastewater characteristics in mg/L	Minimum	Maximum	Mean	Standard deviation	Counts
1	SCOD	12.00	172.67	45.98	24.86	307

#### Predicting SCOD with machine learning

In Machine Learning, the trained models are validated by checking the effluent SCOD prediction on testing dataset and comparing the similarity between actual and predicted values of effluent SCOD. In the present research, effluent SCOD was the target variable, which was predicted based on 6 most important features (inf SCOD, inf CODT, TAlkalinity, TSS, inf NH_3_-N, and inf NO_3_-N) as selected in section "Feature selection".

A series of specialized algorithms is created in machine learning process to recognize the pattern of data, classification, and prediction. The machine learning techniques are quite effective in finding trends in databases that are highly unstructured. In the present study, three algorithms of machine learning, namely ANN, random forest, and decision tree, were used for classification. Traditionally for binary classification, linear regression is utilized as a statistical approach which has become a popular machine learning tool.

In statistics, linear regression refers to a linear method that models the relationship between a scalar response and one or more independent variables. In linear regression, relationships are modeled with linear predictive functions, and the variable parameters of these functions are estimated from the data. In most cases, it is assumed that the conditional mean of the response to the values of the independent variables is an affine map of these values. Other statistical measurements, such as conditional median, were also used. The primary objective of linear regression is conditional probability distribution of the variables. Multivariate analysis, on the other hand, focuses on joint probability distribution.

A decision tree is a sequence model that logically integrates a series of simple tests. In each test, a defined numerical attribute is compared to a set of possible values. As Logical rules used by a decision tree can be easily understood, these symbolic classifiers are more coherent and intelligible than black box models, such as neural networks. Data analysts and decision makers usually prefer an easy-to-understand model. When a data point enters a partitioned area, the decision tree classifies it as the most common class in the area.

Random Forests are sometimes described as Random Decision Forests. It is an ensemble learning technique for classification and regression that uses multiple decision trees and training phases. The mode or mean anticipated value of the results from each decision tree is the output class. To create a single decision tree, a random cell from the given data was chosen. As the association between the individual trees is lessened by randomly choosing the features, random forests have a very high predictive power.

**Figure 6:**
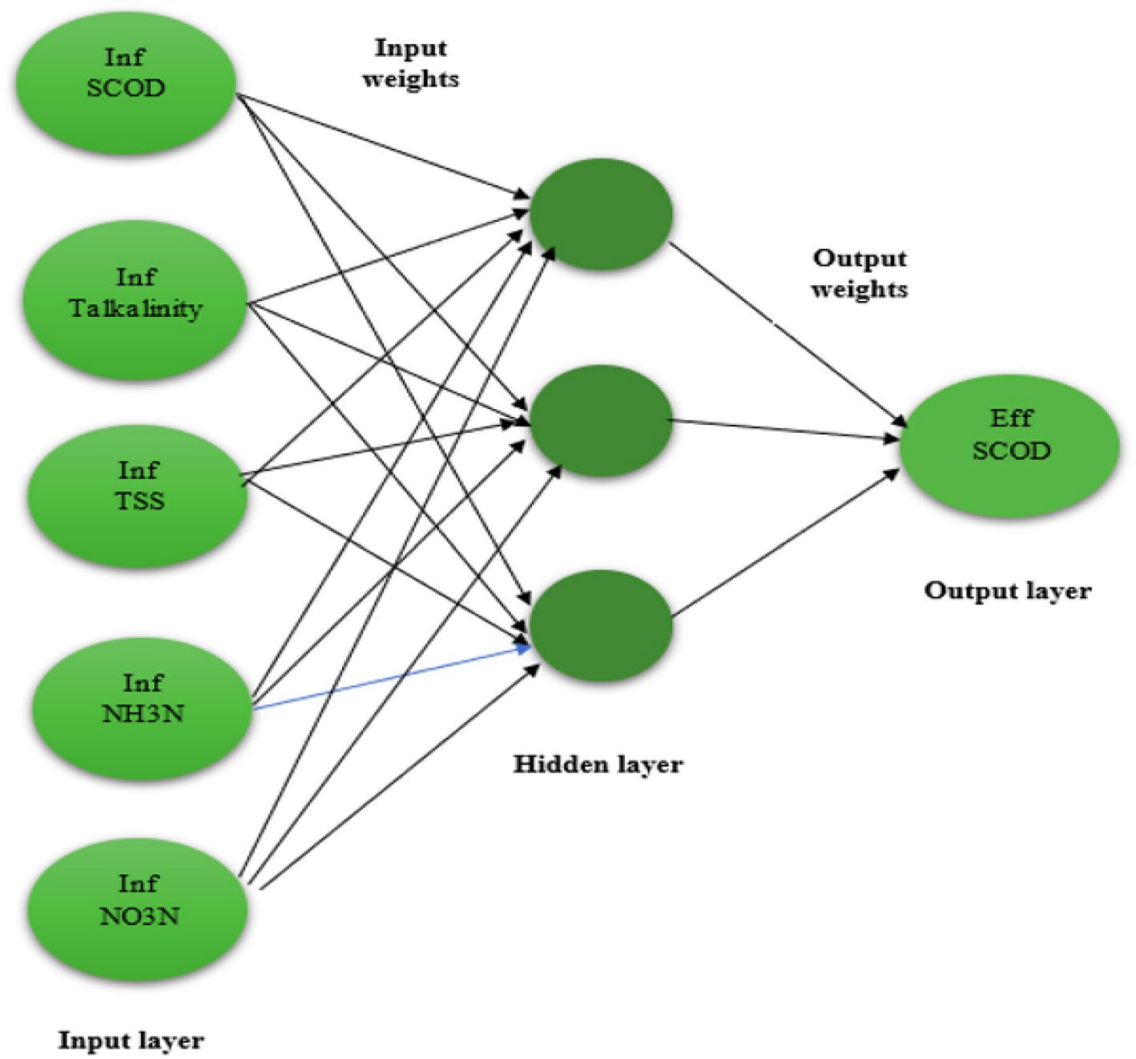
Architecture of neural network.

The ANN is an effective computing tool which ismodelled after the structure and processing capabilities of biological neurons, such as those found in human brain. Similar to human brain, an artificial neural network is made up of simple processing units (called nodes) that interact with one another and process local data. The input signal is received by each node in the network, which then processes it and delivers an output signal to the other nodes. Each node must be connected to at least one other node, and the weight coefficient, a real integer, measures the significance of each connection (synapse). The architecture of the Neural Network topology is demonstrated in Fig. 6.

The input variables in the study and the data collected from influent wastewater are represented by the input neurons. The output and target effluent SCOD is represented by output neuron. With 307 input data points, a 6–1–1 neural network structure was created to train the effluent COD prediction model. The network was trained using a feed-forwardback propagation model, in which a generalised delta rule was used to modify the link weights and biases between the neurons by propagating the mistake at the output neurons backward to the hidden layer neurons and subsequently to the input layer neurons. The study used a tangent sigmoid activation function in the output-layer and a logging transfer function at the hidden layer. The Levenberge-Marquardt backpropagation technique built into the Matlab® Neural Networks Toolbox was used for the training.

## Results and discussion

Anaerobic digestion of wastewater was performed using a two-stage reactor for a period of one year. Parameters of influent water and effluent were recorded. The acquired dataset was used to predict effluent SCOD by various machine learning models. Mean Absolute Percentage error (MAPE) and coefficient of determination (R^2^ score) were adopted as the key performance indicators to authenticate the reliability of the models developed. In statistics, MAPE is a measure of prediction accuracy of a forecasting method for continuous variables. MAPE is calculated by Eq. (1).

1 $$MAPE=\frac{100}{N}\sum_{i=1}^{N}\frac{{A}_{i}-{F}_{i}}{{A}_{i}}$$

Here N is the total number of observations, ${A}_{i}$ is the actual value, and ${F}_{i}$ is the predicted value. R^2^ score denotes the variation in dependent (output) variables, which can be predicted from independent (input) variables. In other words, R^2^ score evaluates the performance of model by checking how well the observed results are reproduced by the model. It can be expressed mathematically by Eq. (2).

2 $${R}^{2}=1-\frac{{SS}_{res}}{{SS}_{tot}}$$

Here ${SS}_{res}$ is the sum of squares of the residual errors and ${SS}_{tot}$ is the total sum of the errors. The value of R^2^ lies between 0 and 1. A forecasting model with R^2^ score close to 1 is considered appropriate.

A 4-plot analysis for all 4 methods is explained here. The primary goal of 4-Plot, a set of four distinct graphical exploratory data analysis (EDA) tools, is to evaluate the assumptions made by the majority of measurement approaches.

### Linear regression

**Figure 7:**
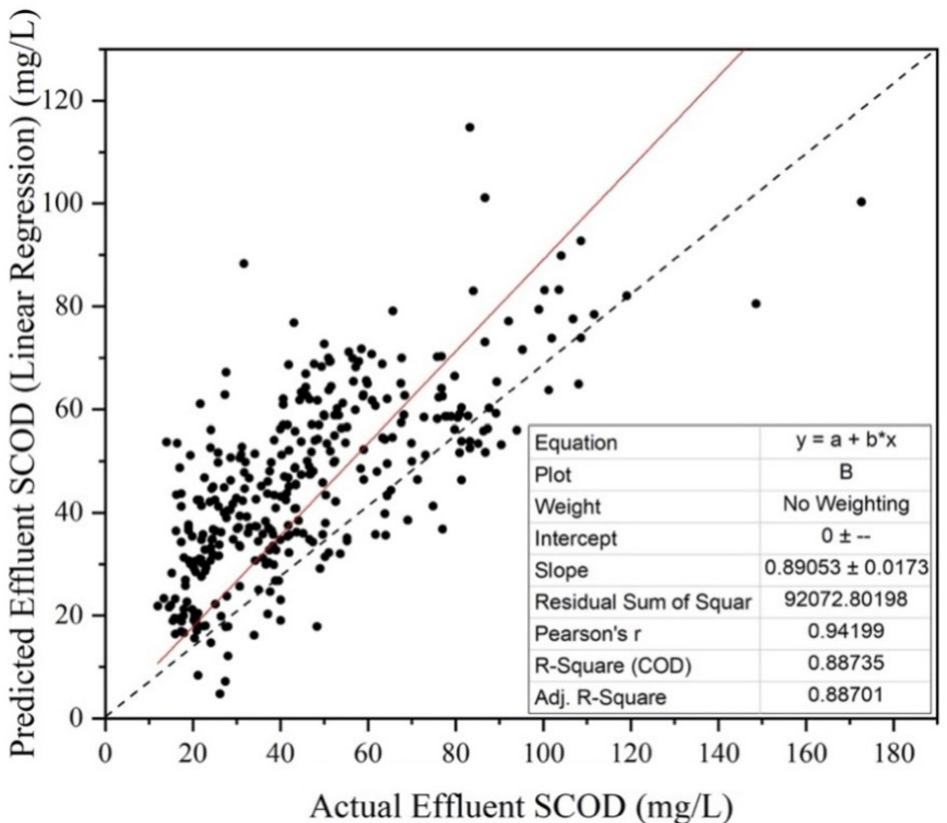
Actual vs predicted values for linear regression.

Figure 7 shows the correlation plot of predicted effluent SCOD vs actual effluent SCOD. The predicted values of effluent SCOD were obtained from Linear Regression. The value of coefficient of determination (R^2^) for the process was obtained to be 0.88. MAPE obtained was 35.87%.

### Decision tree

**Figure 8:**
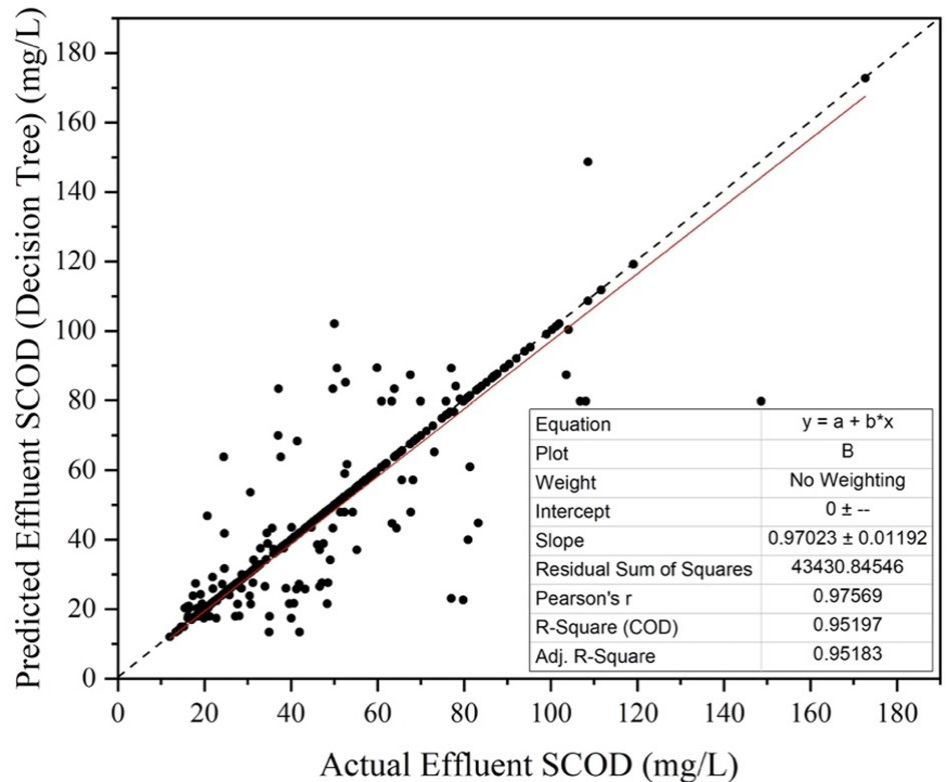
Actual vs predicted values for decision tree.

Decision tree is a machine learning technique in which the data points are split in terms of Decision Nodes and Leaves. In the present study, the actual SCOD measured from the experimental investigation was considered as Leaf, and the various input parameters were considered as Decision Nodes. Figure 8 shows the plot between predicted effluent SCOD and actual effluent SCOD.

From the figure, it can be seen that therewas significant improvement in performance of Decision Tree over Linear Regression. The value of coefficient of determination (R^2^) for the process was obtained to be 0.951. MAPE obtained in this case was 19.23%.

### Random forest

**Figure 9:**
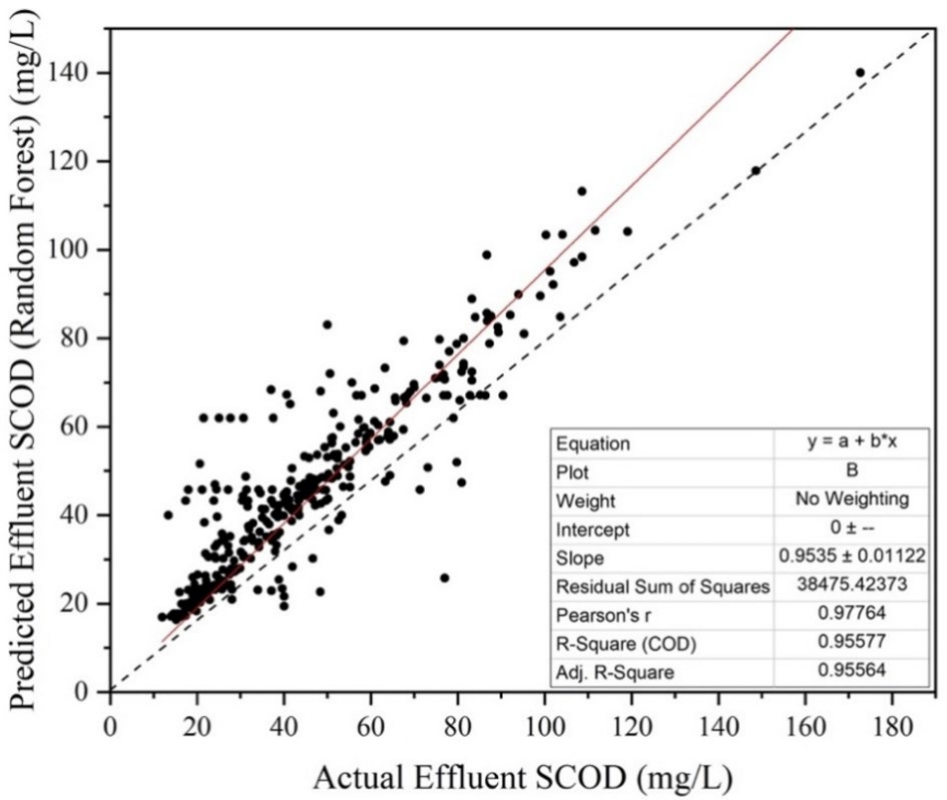
Actual vs predicted values for random forest.

Random forest is one of the most effective machine learning tools that combines several Decision Trees. The technique is more rigorous and takes more time. It compiles randomized decisions based on a number of decisions and bases the final choice on the majority of those decisions. The accuracy of the predicted model depends on the number of trees used in the process. The two steps of the process involve creation of a Random Forest by a number of Decision trees and finally predicting the output from each tree to get the best result. The plot of Predicted Effluent SCOD versus Actual Effluent SCOD is shown in Fig. 9.

The value of coefficient of determination (R^2^ = 0.955) proves that this machine learning technique is slightly better over Decision tree. The value of MAPE obtained in this case was 17.83%.

### Artificial neural network

**Figure 10:**
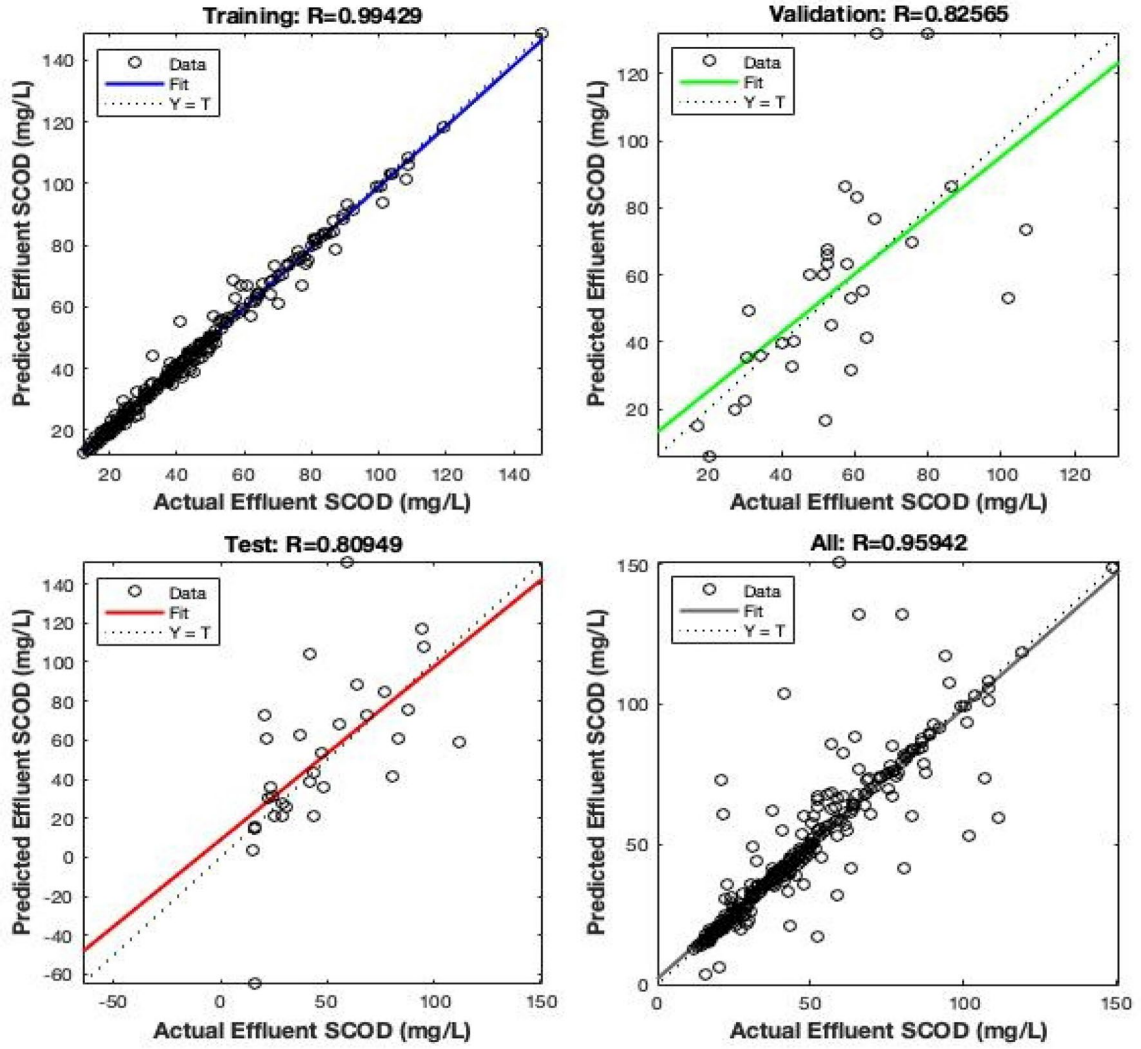
Training, testing and validation of effluent SCOD using neural networks.

The prediction results obtained from Artificial Neural Networks are shown in Fig. 10, 75% of the data points when used to the Train the neural network, 15% to Validate, and 15% to Test yielded the most optimum coefficient of determination. The value of R^2^ obtained for training, validating, and testing were 0.99, 0.82 and 0.80, respectively.

The plot presented in Fig. 10 also shows the predicted effluent SCOD vs actual effluent SCOD (All), whose value of coefficient of determination (R^2^) was 0.959, slightly better over Random Forest. The value of MAPE obtained in this case was observed as 10.63%. The summary of the MAPE values of different machine learning techniques is mentioned in Table 4.

**Table 4:** MAPE values of machine learning techniques adopted in the study.

S/no	Model	MAPE
1	Linear regression	35.87
2	Decision tree	19.23
3	Random forest	17.83
4	Artificial neural networks	10.63

The outcomes demonstrate that the developed method was a quick and useful technique for evaluating the effectiveness of wastewater treatment systems. The ML models implemented in the present study made it possible to use the model structure to successfully predict the dynamic development of the process.

**Figure 11:**
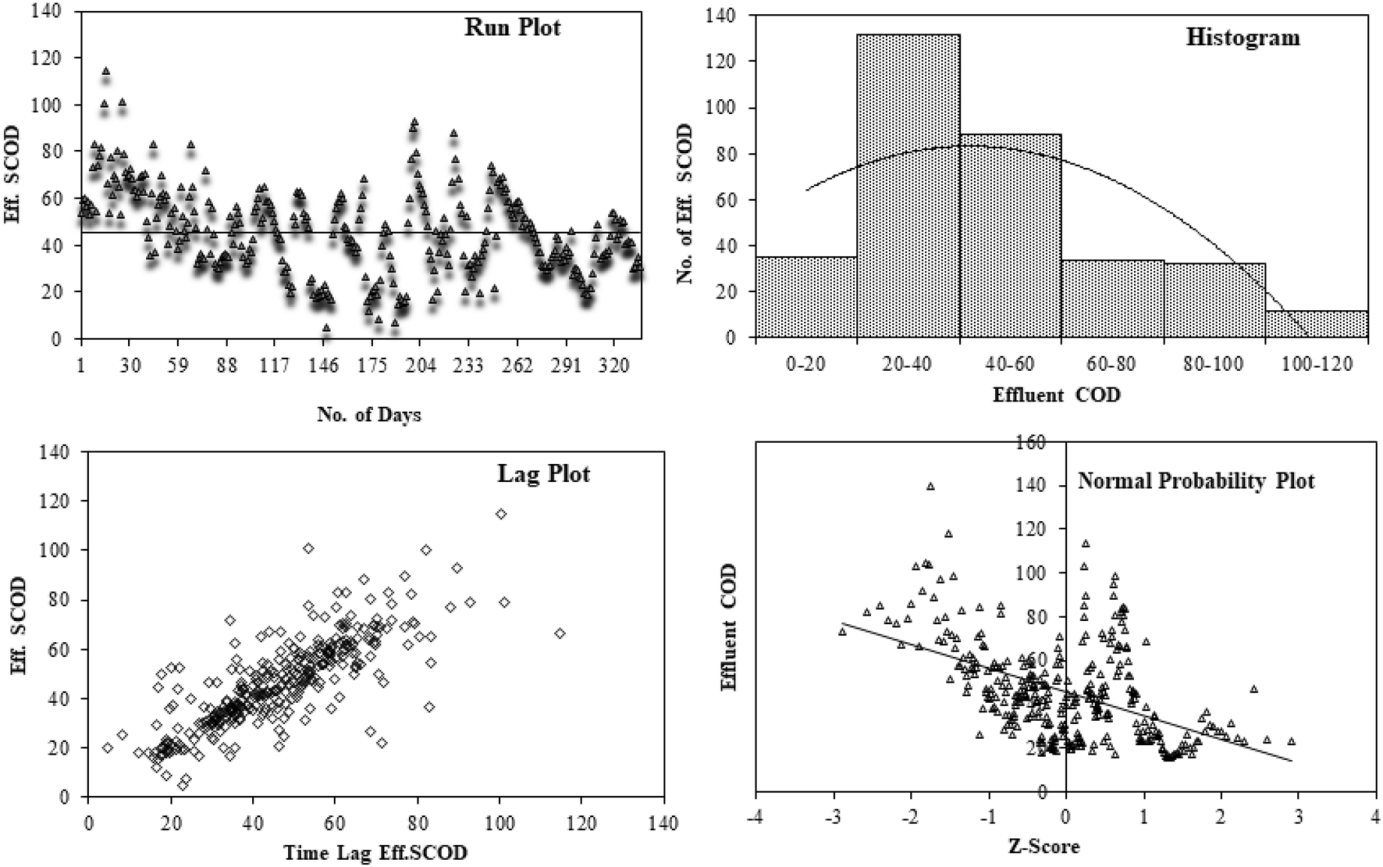
4-plot analysis for ANN.

The 4 plots for ANN, as shown in Fig. 11, represent that:

- There is no drift, and the run sequence plot is straight. The fixed location assumption is thus valid.
- The vertical distribution is likewise fairly comparable in the run sequence plot. As a result, the fixed variation hypothesis is true.
- There are no non-random patterns visible in the lag plot. We can therefore presume that the distribution is random.
- The bell-curve distribution is produced by the histogram. The process is therefore normally distributed.
- In fact, the normal probability plot, which is approximately linear can verify the aforementioned statement.

It shows that the process is "statistically in control" and hence ANN gives best results.

The proposed method is simple in comparison with ADM1 models. The process of anaerobic digestion includes 19 process rate equations, 6 acid–base equilibrium rate equations, 3 gas transfer rate equations, suppression equilibriums, and 32 liquid phase equations for soluble and solid particles. Additionally, ADM1 models require additional 11 kinetic parameters for each metabolic process. However, the proposed machine learning models are data-driven and can predict the evolution of variables without knowing the exact metabolic and dynamic processes or states of the system.

Systematic variable reduction technique was applied using correlation coefficients. The effective selection of variables enabled the machine learning models to effectively predict the SCOD of wastewater. Therefore, the developed method is a fast and reliable estimation method that can be used to predict the SCOD of wastewater to identify the most important process variables. The proposed approach can be used for controlling the influent characteristics. The methodology can be further extended to identify the best substrate compositions. Process variables identified as important can be directly used to predict process performance related to biogas production, so that the biogas production process can be analyzed quickly and efficiently with minimum resources.

## Conclusions

The main objective of the study was to develop and evaluate data driven machine learning models to predict the Effluent SCOD of wastewater. Following conclusions were drawn from the study:

- The advantage of machine learning models over traditional models is due to the fact that Machine Learning models are data driven and can be trained without much knowledge about process kinetics.
- In Machine learning, most significant features were selected by correlation analysis. Feature selection is important to reduce dimensionality without compromising with performance.
- In the present study, influent TKN and influent CODT were 95% correlated. One can be assessed if the other parameter is known. Hence, for the study, influent TKN was omitted, and influent COD was considered.
- Based on the values of coefficient of determination, Artificial Neural Networks had an edge over Random Forest and Decision Tree. The value of R^2^ for ANN, Random Forest, and Decision Tress were 0.959, 0.955 and 0.951, respectively. Linear Regression did not perform well in predicting the Effluent SCOD. The value of R^2^ for Linear Regression was 0.88.
- In terms of Mean Absolute Percentage Error, Artificial Neural Networks performed better than the other three machine learning tools. The value of MAPE for ANN was found to be 10.63% which lowest than other machine learning techniques Lowest MAPE value indicates that ANN gives more accurate prediction results.
- The proposed approach is useful in determining the most important variables for wastewater characterization and their predicted values, and thereby decreasing operations and maintenance cost and time.
- Accordingly, the feed organization and characteristic parameters can be assessed by the design engineer using the fundamental models to improve the cycle yield. Overall, the machine learning models provide a simple approach for forecasting the intricate procedures of wastewater treatment plants.

## Data Availability

The datasets used and/or analyzed during the current study available from the corresponding author on reasonable request.
